# The beneficial effects of a muscarinic agonist on pancreatic β-cells

**DOI:** 10.1038/s41598-019-52691-8

**Published:** 2019-11-07

**Authors:** Yuzuru Ito, Mitsuyo Kaji, Eri Sakamoto, Yasuo Terauchi

**Affiliations:** Department of Endocrinology and Metabolism, Yokohama City University Graduate School of Medicine, Yokohama, Japan

**Keywords:** Hormone receptors, Diabetes

## Abstract

The brain and nervous system play an important role in pancreatic β-cell function. This study investigated the role of muscarinic agonists or acetylcholine, which is the major neurotransmitter in the vagal nerve, in regulating pancreatic β-cell mass and glucose homeostasis. Administration of the muscarinic agonist bethanechol increased insulin secretion and improved glucose tolerance in insulin-receptor substrate 2 (IRS2)-knockout (*IRS-2*^−/−^) mice and diet-induced obesity mice. Oral administration of bethanechol increased β-cell mass and proliferation in wild-type mice, but not *IRS-2*^−/−^ mice. The muscarinic agonist also increased the incorporation of 5-bromo-2′-deoxyuridine (BrdU) into islets isolated from wild-type mice and pancreatic β-cell line MIN6. The phosphorylation of protein kinase B (Akt) induced by oral administration of bethanechol was observed in wild-type mice, but not *IRS-2*^−/−^ mice. The secretion of glucagon-like peptide-1 (GLP-1) was also stimulated by bethanechol in wild-type mice, and a GLP-1 antagonist partially inhibited the bethanechol-induced increase in β-cell mass. These results suggest that the muscarinic agonist exerted direct and indirect effects on β-cell proliferation that were dependent on the IRS-2/Akt pathway. The bethanechol-stimulated release of GLP-1 may be indirectly associated with β-cell proliferation.

## Introduction

The brain and the nervous system are well recognized for their important role in metabolic control. Previous studies have demonstrated how the nervous system regulates glucose homeostasis and contributes towards pancreatic β-cell regeneration^1–3^. In obese and hyperglycaemic ob/ob mice, glucose tolerance and islet proliferation rates were analysed following vagotomy^1^. It was observed that in vagotomised mice, the islet cell proliferation rate was reduced, and the islet volume was smaller. Another study reported that vagal hyperactivity, which can occur as a result of ventromedial hypothalamic lesions, induced pancreatic β-cell proliferation^2^. Moreover, it was reported that the activation of obesity-related hepatic extracellular signal-related kinase (ERK) caused the proliferation of pancreatic β-cells through signal transduction to the nervous system^3^. These results suggest that the vagal nerve system affects the proliferative ability of pancreatic β-cells. In this study, we aim to elucidate the neurotransmitters or receptors that are responsible for increasing the β-cell mass following vagal stimulation and determine whether direct or indirect mechanisms are involved in this activity.

Because the muscarinic receptor agonist acetylcholine is the main neurotransmitter of vagal nerve signals and M3 muscarinic receptor is predominant in the β-cell, several studies have reported the relationship between the M3 muscarinic receptor and pancreatic β cell maintenance. The results are described as follows: (1) Acetylcholine releases insulin in a glucose-dependent manner in the β-cell; (2) The continuous activation of the M3 receptor in transgenic mice improved glucose homeostasis and increased insulin release^4^, and β-cell-specific knockout mice for the M3 muscarinic receptor decreased insulin release and impaired glucose tolerance^5^; (3) The continuous activation of the M3 designer receptor improved glucose tolerance and increased pancreatic β-cell through the IRS2/Akt pathway^6^ and (4) The co-stimulation of carbachol and pituitary adenylate cyclase-activating polypeptide (PACAP) increased β-cell proliferation via FoxM1 in isolated islets^7^. Because adult β-cells were recently reported to be maintained by self-replication from the existing β cells in the islets^8,9^, the muscarinic receptor signal increasing the adult β-cell replication for the regeneration of cells in individuals with diabetes mellitus could be a potential therapeutic target.

The present study aimed to elucidate the proliferative mechanism or effect of the muscarinic receptor which remains unclear, *in vivo* or in islets. In this study, we used bethanechol as a muscarinic agonist that can be administered *in vivo*. Our results indicated that the muscarinic agonists increased β-cell mass and proliferation. In addition, we investigated the potential involvement of the insulin signal molecules IRS-2/Akt or GLP-1 release in muscarinic agonist-induced pancreatic β-cell proliferation. These molecules are involved in the maintenance of pancreatic β-cell mass^10–17^.

## Results

### Administration of muscarinic agonist bethanechol improves glucose homeostasis in diabetic model mice and diet-induced obesity mice

**Figure 1:**
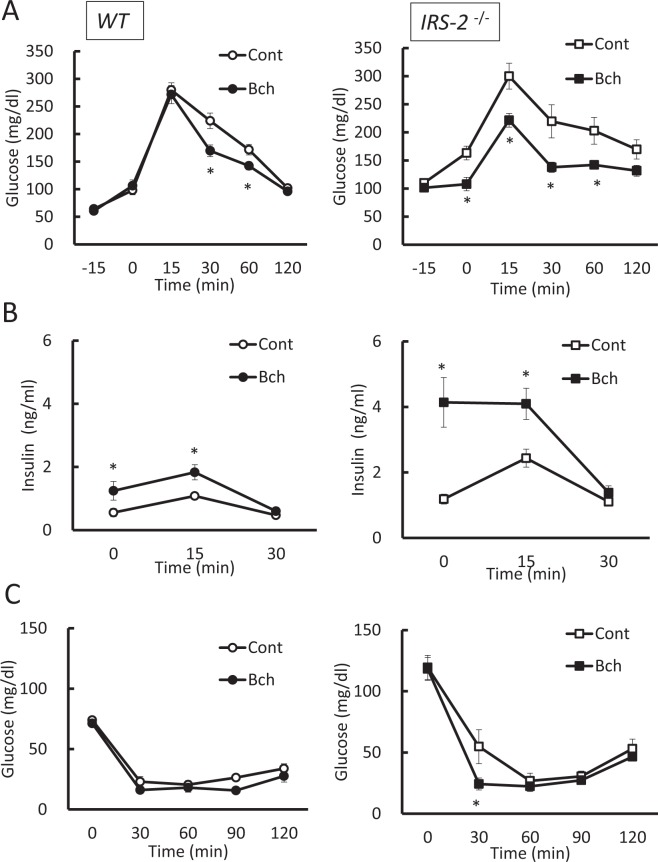
Administration of bethanechol improves glucose tolerance in a mouse model of diabetes. Experiments were performed in 8- to 10-week-old wild-type (WT) mice and insulin-receptor substrate 2 (*IRS-2*)^−/−^ mice in standard chow diet–fed condition. (**A**) Plasma glucose levels during the oral glucose tolerance test (OGTT) following administration of bethanechol (Bch; 2 µg/g body weight). Bch was administered subcutaneously 15 min prior to oral glucose loading (1.5 mg/g body weight) in WT mice (left; n = 10) and *IRS-2*^−/−^ mice (right; n = 11). (**B**) Serum insulin levels during the OGTT following administration of Bch (2 µg/g) in WT mice (left; n = 10) and IRS*-2*^−/−^ mice (right; n = 10). (**C**) Plasma glucose levels during the insulin tolerance test following administration of Bch (2 µg/g). Bch was administered subcutaneously 15 min prior to insulin injection in WT mice (left; n = 6) and *IRS-2*^−/−^ mice (right; n = 4). The data represent the mean ± standard error of the mean (SEM); *P < 0.05 vs. control.

To examine the effect of bethanechol in a mouse model of diabetes, it was administered to *IRS-2*^−/−^ mice, which show insulin resistance and high glucose levels. Bethanechol (2 µg/g) was injected subcutaneously 15 min prior to oral glucose loading in wild-type mice and *IRS-2*^−/−^ mice. Bethanechol increased insulin secretion and improved glucose tolerance in both types of mice (Fig. 1A). The bethanechol-induced secretion of insulin in the *IRS-2*^−/−^ mice was higher than that in the wild-type mice (Fig. 1B). The results of the insulin tolerance test revealed no significant difference in glucose levels between the control and bethanechol groups of wild-type mice; however, 30 min following insulin injection, the glucose level in the bethanechol-injected *IRS-2*^−/−^ mice was significantly lower than that in the control mice (Fig. 1C).

**Figure 2:**
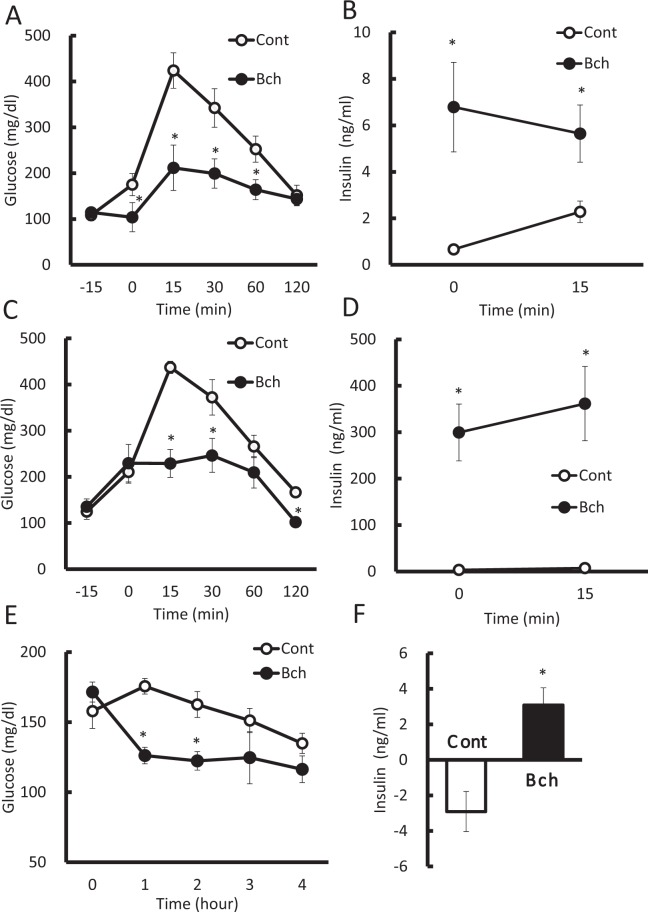
Administration of bethanechol (Bch) improves glucose tolerance and casual glucose in diet-induced obesity mice. A high-fat diet (HFD) was started in 7-week-old C57BL/6J mice and experiments were performed for 5–6 or 16 weeks following HFD feeding. (**A**) Plasma glucose levels and (**B**) serum insulin levels in the oral glucose tolerance test (OGTT) following administration of Bch (5 µg/g body weight) in HFD-fed mice for 6 weeks. Bch was administered subcutaneously 15 min prior to oral glucose loading (1.5 mg/g body weight) (n = 5–6). (**C**) Plasma glucose levels and (**D**) serum insulin levels in the OGTT following administration of Bch (5 µg/g) in HFD-fed mice for 16 weeks. Bch was administered subcutaneously 15 min before oral glucose loading (1.5 mg/g body weight) (n = 5–6). (**E**) Casual glucose levels and (**F**) serum insulin levels following oral administration of Bch (100 µg/g). Bch was administered orally and HFD was stopped for 4 h (n = 5–6). (**F**) Data are expressed as the increase from the 0-h insulin level to the 1-h insulin level. The data represent the mean ± standard error of the mean (SEM); *P < 0.05 vs. control.

To examine the effect of bethanechol on diet-induced obesity (DIO) mice, 7-week-old male mice were fed a high-fat diet (HFD) for 5–6 or 16 weeks, following which an oral glucose tolerance test was performed. The subcutaneous administration of bethanechol (5 µg/g) prior to oral glucose loading improved glucose tolerance (Fig. 2A,C) and increased insulin secretion markedly (Fig. 2B,D). Oral administration of bethanechol (100 µg/g) also improved casual glucose after 1 and 2 h of administration (Fig. 2E), and increased insulin secretion significantly after 1 h in DIO mice (Fig. 2F).

Bethanechol administration caused cholinergic side effects in DIO mice and wild-type (WT) mice. The number of mice exhibiting salivation or lacrimation was counted. Subcutaneous administration of bethanechol (5 µg/g) in the mice fed a HFD for 5–6 or 16 weeks, respectively, induced salivation (41.6% and 100%) and lacrimation (25% and 33.3%) (Supplementary Table). In the mice administered oral bethanechol (100 µg/g), no salivation or lacrimation were observed. Bethanechol (5 µg/g) administered subcutaneously also caused miosis in WT mice (slight contraction from 4 mm to 3–3.5 mm), as measured macroscopically with a loupe (data not shown).

### Chronic administration of bethanechol promotes β-cell proliferation

**Figure 3:**
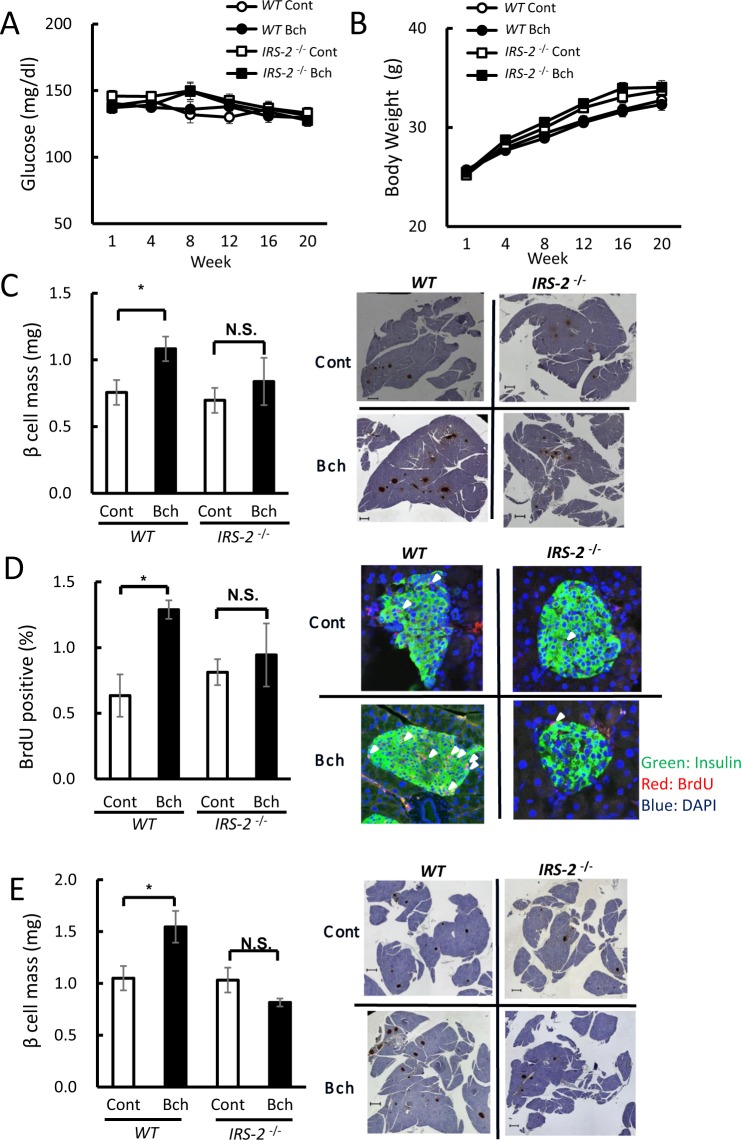
Chronic administration of bethanechol (Bch) promotes ß-cell proliferation mediated by insulin-receptor substrate 2 (IRS-2). Eight-week-old wild-type (WT) mice and *IRS-2*^−/−^ mice were administered Bch (30 µg/mL) in drinking water. (**A**) Casual blood glucose level and (**B**) body weight in WT mice and *IRS-2*^−/−^ mice for 20 weeks (n = 12–15). (**C**) Quantification of the β-cell mass in WT mice and *IRS-2*^−/−^ mice administered Bch for 6 weeks (left; n = 6–8), and representative pancreatic sections (right). (**D**) Quantification of 5-bromo-2′-deoxyuridine (BrdU)-positive and insulin-positive cells in WT mice and *IRS-2*^−/−^ mice administered Bch for 6 weeks (left). Insulin is stained green, nuclei are stained blue and BrdU-positive nuclei are stained red. The white arrows indicate BrdU-positive, insulin-positive cells (right). (**E**) Oral administration of Bch promoted β-cell proliferation. Eight-week-old WT mice and *IRS-2*^−/−^ mice were orally administered Bch 1 µg/g once a day for 2 weeks (n = 4). Quantification of the β-cell mass in WT mice and *IRS-2*^−/−^ mice (left), and representative pancreatic sections (right). The data represent the mean ± standard error of the mean (SEM); *P < 0.05 vs. control.

Long-term administration of bethanechol in mice was examined by measuring blood glucose levels and changes in body weight. Bethanechol was added to drinking water at a concentration of 30 µg/mL, and the casual blood glucose and body weight were measured for 20 weeks in WT and *IRS-2*^−/−^ mice. There were no significant differences in body weight, casual blood glucose or glucose tolerance between mice with or without bethanechol administration, in both WT and the *IRS-2*^−/−^ mice (Figs 3A,B; S1). The β-cell mass of mice continuously administered bethanechol (30 µg/mL in water) was also evaluated for 6 weeks. In the WT mice, the β-cell mass increased significantly in those receiving bethanechol compared with the controls (control: 0.76 mg; bethanechol: 1.08 mg). However, there was no significant difference in the β-cell mass between the bethanechol-administered *IRS-2*^−/−^ mice and control *IRS-2*^−/−^ mice (control: 0.70 mg; bethanechol: 0.84 mg; Fig. 3C).

The incorporation of 5-bromo-2′-deoxyuridine (BrdU) was assessed to determine the effect of the muscarinic agonist on the proliferation of β-cells. As shown in Fig. 3D, bethanechol (30 µg/mL in water) administration increased the number of BrdU-positive cells (control: 0.63%; bethanechol: 1.29%) in the WT mice. In the *IRS-2*^−/−^ mice, no significant increase in BrdU-positive cells was observed following agonist administration (control: 0.81%; bethanechol: 0.94%; Fig. 3D).

To examine the issue of bethanechol stability in water, bethanechol was given orally at 1 µg/g body weight once a day, and the β-cell mass was measured 2 weeks following the start of administration (Fig. 3E). The WT mice that were treated with bethanechol exhibited an increase in the β-cell mass compared with control mice (control: 1.05 mg; bethanechol: 1.55 mg), whereas no significant increase was observed in the *IRS-2*^−/−^ mice (control: 1.03 mg; bethanechol: 0.82 mg).

### Bethanechol directly stimulates pancreatic β-cell proliferation

**Figure 4:**
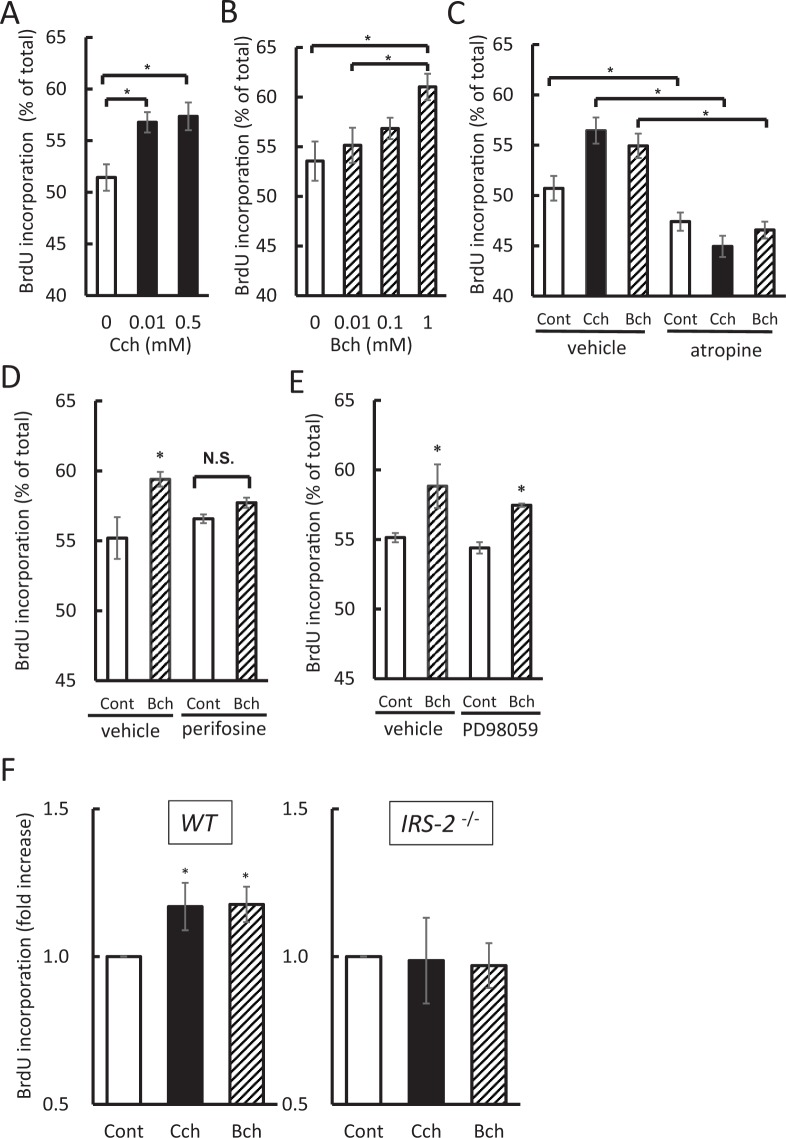
Bethanechol (Bch) directly promotes β-cell proliferation. (**A**–**E**) MIN6 cells were treated with Bch (1 mM) or carbachol (Cch; 0.5 mM) for 24 h. Cells were simultaneously incubated with the agonist and atropine (50 µM), perifosine (5 µM) or PD98059 (50 µM). 5-Bromo-2′-deoxyuridine (BrdU) was then added to the media and the cells were fixed 20 h after the addition of BrdU. BrdU-positive cells were measured using flow cytometry. (**F**) Isolated islets from 8-week-old wild-type (WT) mice and insulin-receptor substrate 2 (*IRS-2*) ^−/−^ mice were incubated in media containing Bch (1 mM) or Cch (0.5 mM) and BrdU for 24 h. The islets were fixed and BrdU incorporation was measured using a BrdU cell proliferation assay kit. (**A**) Cch-induced BrdU incorporation in MIN6 cells (n = 10). (**B**) Bch-induced BrdU incorporation in MIN6 cells (n = 6). (**C**) Effect of atropine (50 µM) on Bch- or Cch-induced BrdU incorporation in MIN6 cells (n = 6). (**D**) Effect of perifosine (5 µM) on Bch-induced BrdU incorporation in MIN6 cells (n = 4). (**E**) Effect of PD98059 (50 µM) on Bch-induced BrdU incorporation in MIN6 cells (n = 4). (**F**) Bch- or Cch-induced BrdU incorporation in isolated islets from WT mice (left; n = 23) and *IRS-2*^−/−^ mice (right; n = 8). The data represent the mean ± standard error of the mean (SEM); *P < 0.05 vs. control.

To determine whether bethanechol has a direct effect on pancreatic β cells, BrdU incorporation was investigated in the pancreatic β-cell line MIN6. MIN6 cells were cultured in media containing BrdU and the muscarinic agonist for 24 h, following which the cells were fixed, stained with fluorescent isothiocyanate (FITC)-labelled BrdU antibodies and analysed by flow cytometry. BrdU incorporation increased significantly in the carbachol-treated or bethanechol-treated cells in a concentration-dependent manner (Fig. 4A,B). This increase was suppressed completely by the addition of atropine 50 µM (Fig. 4C). The Akt inhibitor perifosine (5 µM) inhibited the bethanechol (1 mM)-induced incorporation of BrdU (Fig. 4D), whereas the mitogen-activated protein kinase (MEK) inhibitor PD98059 (50 µM) did not (Fig. 4E).

Islets were isolated from 8-week-old WT or *IRS-2*^−/−^ mice to examine the incorporation of BrdU following treatment with muscarinic agonists in a similar manner. Islets were incubated in media containing bethanechol (1 mM) or carbachol (0.5 mM) and BrdU for 24 h, after which BrdU incorporation was measured. Similar to that in MIN6 cells, a significant increase in BrdU incorporation was observed following the stimulation of islets from WT mice with bethanechol (1 mM) or carbachol (0.5 mM) (Fig. 4F). In islets from the *IRS-2*^−/−^ mice, no BrdU incorporation was observed (Fig. 4F). Collectively, these results suggest that muscarinic agonists directly affected the proliferative ability of pancreatic β-cells through IRS-2.

### Phosphorylation of Akt and protein level of cyclin D2 are increased in bethanechol-treated mouse islets

**Figure 5:**
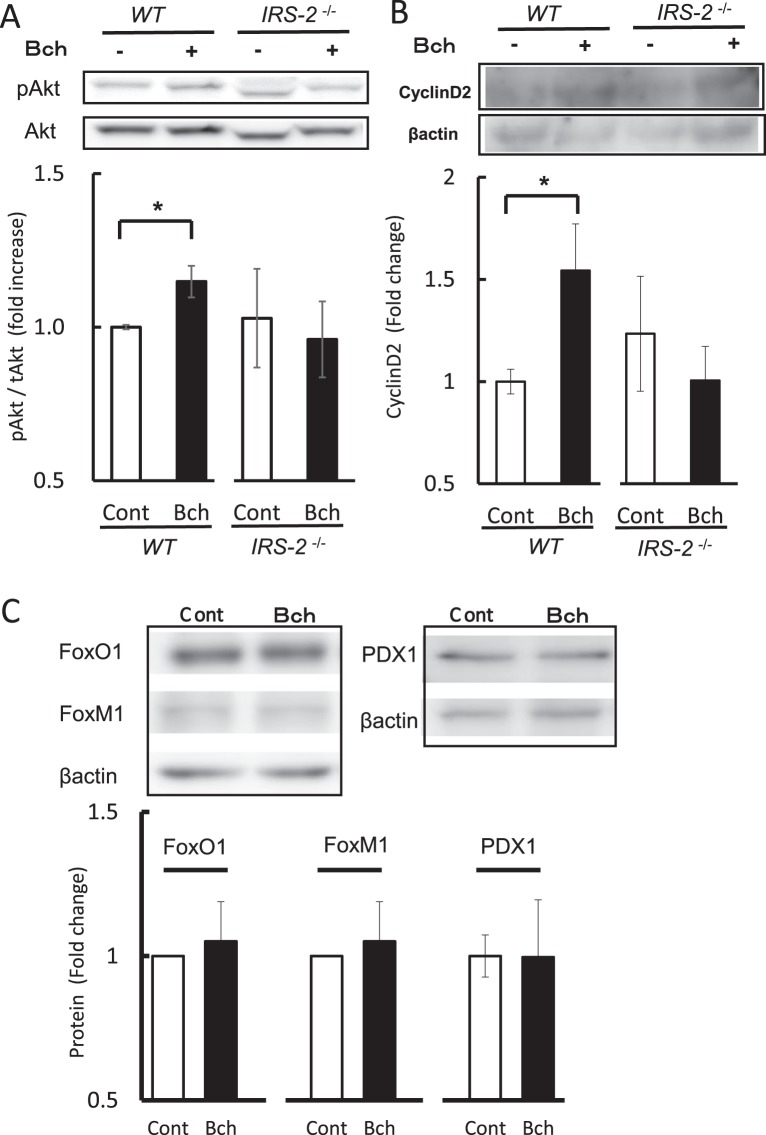
The muscarinic agonist increases the phosphorylation of Akt and expression of cyclin D2 protein in mouse islets. (**A**) Phosphorylation of Akt and (**B**) expression of cyclin D2 protein were evaluated in isolated islets from wild-type (WT) mice and insulin-receptor substrate 2 (*IRS-2*)^−/−^ mice orally administered bethanechol (Bch; 1 µg/g body weight) once a day for 1 week (n = 4–9). (**C**) Protein levels of FoxO1, FoxM1 and PDX1 were analysed in WT mice administered Bch (1 µg/g) once a day for 1 week (n = 3–7). The data represent the mean ± standard error of the mean (SEM). *P < 0.05 vs. control.

To assess the mechanisms involved in muscarinic agonist-mediated β-cell proliferation, intracellular signalling was investigated in mouse islets. Following oral administration of bethanechol (1 µg/g) for 1 week in WT and *IRS-2*^−/−^ mice, islets were isolated and the phosphorylation of Akt was analysed by western blotting. The phosphorylation of Akt increased significantly in response to bethanechol in the WT mice, but not in the *IRS-2*^−/−^ mice (Fig. 5A). The protein level of cyclin D2 also increased in response to bethanechol in the WT mice, but not in the *IRS-2*^−/−^ mice (Fig. 5B). Bethanechol did not alter the protein levels of transcription factors FoxO1, FoxM1 or PDX1 (Fig. 5C). These results suggest that the IRS-2/Akt/cyclin D2 pathway mediated bethanechol-induced β-cell proliferation.

### GLP-1 is released in bethanechol-treated mice and its antagonist inhibits the bethanechol-induced increase in β-cell mass

**Figure 6:**
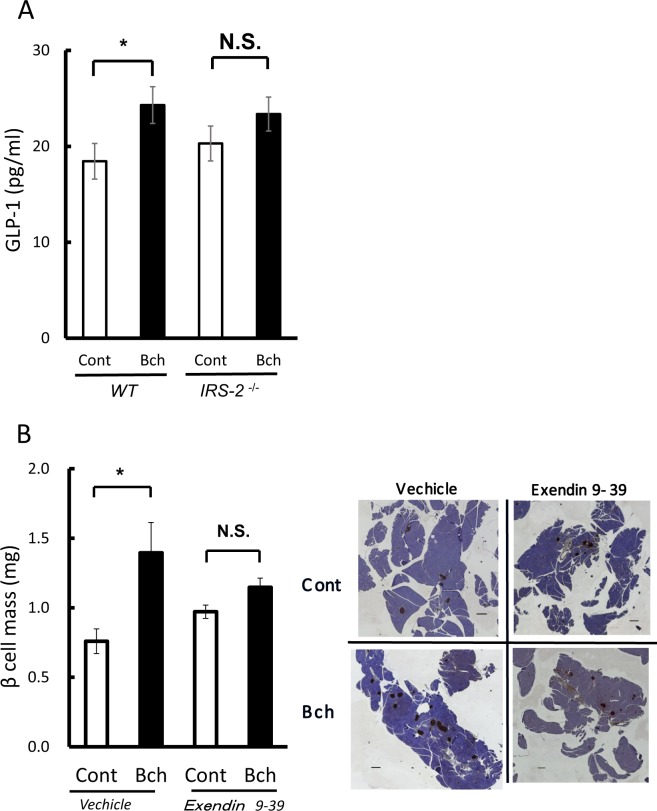
Bethanechol (Bch) increases the secretion of glucagon-like peptide-1 (GLP-1) and pretreatment with the GLP-1 antagonist suppresses the Bch-induced increase in β-cell mass in mice. (**A**) Eight-week-old C57BL/6J mice were starved for 18–22 h and Bch (2 µg/g body weight) was subcutaneously administered to wild-type (WT) mice and insulin-receptor substrate 2 (*IRS-2*)^−/−^ mice (n = 17–19). Serum GLP-1 levels were measured 15 min after Bch administration. (**B**) Pretreatment with exendin 9–39 suppressed the increase in β-cell mass caused by Bch in C57BL/6J mice. Eight-week-old C57BL/6J mice were administered Bch (10 µg/g) orally for 2 weeks. Exendin 9–39 (30 pmol/g body weight) was intraperitoneally administered 15 min prior to Bch administration. The β-cell mass was quantified (left; n = 4), with representative pancreatic sections (right). The data represent the mean ± standard error of the mean (SEM). *P < 0.05 vs. control.

To analyse the indirect effects of bethanechol, we focused on glucagon-like peptide-1 (GLP-1). The secretion of GLP-1 was measured 15 min following the subcutaneous administration of bethanechol (2 µg/g). GLP-1 secretion increased significantly in the bethanechol-treated WT mice, but there was no significant change in the secretion of GLP-1 in bethanechol-treated *IRS-2*^−/−^ mice (Fig. 6A).

To investigate the role of GLP-1, the mice were pretreated with the GLP-1 antagonist exendin 9–39 (30 pmol/g, intraperitoneally), 15 min prior to oral administration of bethanechol. Bethanechol was added at 10 µg/g once a day and the β-cell mass was measured 2 weeks following the start of administration. Bethanechol (10 µg/g) administration increased the β-cell mass significantly in the vehicle mice (control: 0.76 mg; bethanechol: 1.39 mg), whereas no significant increase in the β-cell mass was observed following agonist administration in the mice pretreated with exendin 9–39 (control: 0.97 mg; bethanechol: 1.15 mg) (Fig. 6B). However, exendin 9–39 did not completely suppress the increase in β-cell mass in the bethanechol-treated mice; there was still a bethanechol-induced marginal increase in the β-cell mass compared with that in the controls.

## Discussion

In mice, islets are largely dominated by parasympathetic neurons^18^. Generally, in the pancreatic islets, the parasympathetic neurons promote insulin secretion and the sympathetic neurons inhibit insulin secretion^19–21^; the brain and the vagal nervous system play an important role in pancreatic β-cell proliferarion. Because acetylcholine is the major neurotransmitter stored in vagal nerve endings, we aimed to elucidate the association between muscarinic receptor agonists and β-cell maintenance. Our results demonstrated that the muscarinic receptor agonist bethanechol improved glucose homeostasis in *IRS-2*^−/−^ and DIO mice, and increased the pancreatic β-cell proliferation. These proliferative effects were caused via simultaneous activation of the muscarinic receptor and the GLP-1 receptor signal (Fig. S2).

Because vagal hyperactivity induces pancreatic β-cell proliferation, we hypothesised that chronic activation of muscarinic receptors in pancreatic β-cells might increase the β-cell mass. To test this hypothesis, we subjected mice to continuous, chronic administration of bethanechol, which significantly increased β-cell mass and BrdU incorporation (Fig. 3C–E). Furthermore, bethanechol-induced incorporation of BrdU was elevated significantly in islets isolated from the mice (Fig. 4F) and MIN6 cells (Fig. 4B), suggesting that this muscarinic agonist directly affects the proliferative ability of pancreatic β-cells.

We confirmed that the M1 and M3 muscarinic receptors were expressed, and the M3 receptor was predominant in the murine β-cells (Fig. S3). The β-cell mass increased in the transgenic mice with chronic activation of the Gq-coupled designer receptor which was created based on the M3 muscarinic receptor^6^, and mice treated with scopolamine butylbromide (a cholinergic antagonist) during lactation show lowered expression of the M3 receptor and decreased β-cell mass^22^. These findings suggest that the Gq-signalling of the M3 receptor was responsible for the β-cell mass expansion. However, a previous investigation on the effect of continuous stimulation of the β-cell M3 receptor on the β-cell mass and replication in transgenic mice demonstrated that it did not have a significant effect on the increase in the β-cell mass^4^. Pancreatic β-cell-specific knockout mice for the M3 muscarinic receptor do not exhibit obvious differences in the β-cell mass and cell size compared with WT mice^23^. Therefore, the direct effect of bethanechol on β-cell proliferation could not be caused by the M3 receptor signal alone. Because bethanechol activates all muscarinic receptor subtypes, activation of other muscarinic Gq receptor (e.g. M1) in the β-cell is also needed in bethanechol-induced β-cell proliferation.

It is well recognised that insulin signal molecules are important in the regulation of β-cell proliferation and mass expansion. Previous reports have suggested that IRS-2 and Akt proteins play a dominant role in regulating β-cell proliferation and maintaining β-cell mass^13–15,24^. Akt1 increases the levels of cyclins D1 and D2, and the cyclin/CDK4/Akt complex is responsible for β-cell proliferation^25^. Gq-coupled receptor-signalling also increases β-cell mass through IRS-2/Akt pathway^6^. To identify the mechanisms of bethanechol-induced β-cell proliferation, we examined the role of insulin signal molecules. Bethanechol did not increase the β-cell mass in the *IRS-2*^−/−^ mice (Fig. 3C,E), and it phosphorylated Akt protein and increased the protein levels of cyclin D2 in an IRS-2-dependent manner (Fig. 5). Our findings suggest that the bethanechol-induced β-cell proliferation was mediated by the IRS-2/Akt/cyclin D2 pathway. Although ERK1 and ERK2 are reported to be upstream of the function of IRS-2 in the Gq-coupled receptor pathway^6^, the MEK inhibitor did not inhibit the bethanechol-induced incorporation of BrdU in MIN6 cells (Fig. 4E). The muscarinic agonist-induced β-cell proliferation might occur independently of ERK. A recent report indicated that co-stimulation of carbachol and PACAP promotes β-cell proliferation via FoxM1 in isolated islets^7^. Whereas we demonstrated that carbachol or bethanechol alone could increase BrdU uptake in islets or MIN6 cells (Fig. 4A,B,F), bethanechol administration did not affect the protein levels of FoxM1 (Fig. 5), and it did not lead to translocation of FoxM1 to the nucleus in murine islets (Fig. S4). It is possible that bethanechol regulates the activity of FoxM1 rather than changing its protein level or localisation.

Besides the direct effects of bethanechol to the β-cell, the indirect mechanisms may also be involved in this bethanechol-induced β-cell proliferation *in vivo*. Bethanechol might increase the secretion of other hormones involved in the regulation of β-cell mass, which include GLP-1, insulin-like growth factors, growth hormone, hepatocyte growth factor, parathyroid hormone-related protein, the epidermal growth factor ligand family and lactogens. It is reported that β-cell proliferation requires Gs-signalling (PACAP receptor), in addition to Gq-signalling (M3 muscarinic receptor)^7^. Muscarinic receptor subtypes M1, M2 and M3 are expressed in the distal intestine^26,27^ and our findings showed that bethanechol enhanced the release of GLP-1 (Fig. 6A), which is known to increase β-cell mass and activate Gs-signalling^16,17^. The GLP-1 antagonist partially suppressed the increase in β-cell mass in the bethanechol-treated mice (Fig. 6B). These results suggest that the GLP-1 receptor pathway is a factor in the muscarinic receptor-independent pathway. Therefore, the effects of the vagal nerve or bethanechol in pancreatic β cells may be exerted through the Gq- and Gs-signalling pathways (Fig. S2).

Moreover, the indirect effects of bethanecol also affect the enhancement of insulin release. We indicated that the subcutaneous administration of bethanechol increased insulin release in the *IRS-2*^−/−^ mice more than that in the WT mice (Fig. 1B), resulting in improved glucose tolerance in the *IRS-2*^−/−^ mice relative to the wild-type mice (Fig. 1A). However, the glucose-stimulated secretion of insulin was similar in the islets of the *IRS-2*^−/−^ and WT mice (Fig. S5). Other unknown endogenous secretory proteins are associated with the enhancement of insulin release in the *IRS-2*^−/−^ mice. To identify these secretory factors in the *IRS-2*^−/−^ mice, we performed mass spectrometry-based proteomics analyses of plasma obtained from the control or *IRS-2*^−/−^ mice; these experiments remain under investigation.

In conclusion, we demonstrated the beneficial effects of bethanechol on glucose homeostasis and pancreatic β-cell maintenance. The administration of bethanechol improved glucose homeostasis, and it directly or indirectly stimulated β-cell proliferation. The GLP-1 receptor pathway may be indirectly associated with β-cell proliferation. Therefore, muscarinic agonists and their downstream molecules could be potential targets for novel diabetic therapies.

## Methods

### Animals and animal care

The C57BL/6J WT mice were obtained from CLEA Japan. We backcrossed *IRS-2*^−/−^ mice^28^ with C57BL/6J mice more than 10 times. All experiments were performed using male mice maintained on a standard chow diet. The chronic effects of bethanechol were investigated in *IRS-2*^−/−^ mice and C57BL/6 J mice. Bethanechol was administered continuously (30 µg/mL in drinking water) or once-daily (1 µg/g given orally) in the *IRS-2*^−/−^ mice and WT mice. In the exendin 9–39 experiment, 30 pmol/g exendin 9–39^29^ was administered intraperitoneally 15 min prior to bethanechol 10 µg/g administration orally in C57BL/6J mice for 2 weeks. In the experiment involving DIO mice, 7-week-old mice were fed a HFD. All animal procedures were performed in accordance with the institutional animal care guidelines and guidelines of the Animal Care Committee of Yokohama City University. The protocol was approved by the Yokohama City University Institutional Animal Care and Use Committee (permit no. F-A-18-020). The animals were housed in rooms maintained at a constant room temperature (25 °C) and on a 12-h light (from 07:00 AM onwards) and 12-h dark (from 7:00 PM onwards) cycle^30^.

### Glucose tolerance and insulin tolerance tests

The plasma glucose levels and blood insulin levels were determined using GlutestNeoSuper (Sanwa Chemical Co.,) and an insulin kit (Morinaga), respectively. All mice were fasted for 18–22 hours prior to the oral glucose tolerance test followed by oral administration of glucose (1.5 mg/g body weight). An insulin tolerance test was performed by the intraperitoneal injection of mice with human insulin (0.75 mU/g body weight)^31^.

### GLP-1 measurement

All mice were denied access to food for 18–22 h prior to subcutaneous administration of bethanechol (2 µg/g body weight). The active GLP-1 levels were measured 15 min after bethanechol administration using a GLP-1(Active) ELISA kit (Shibayagi). Blood was drawn into a syringe containing aprotinin (final concentration, 500 kIU/mL; Wako Pure Chemical Industries, Ltd.) as a dipeptidyl peptidase-IV inhibitor.

### Histologic analysis

Mice were intraperitoneally injected with BrdU (100 µg/g; Nacalai Tesque, Inc) for 3 days and sacrificed. Three pancreatic tissue sections (100 µm apart) from each animal were prepared by fixation and paraffin-embedding. The sections were immunostained with antibodies to insulin, muscarinic acetylcholine receptor M1 (Santa Cruz), BrdU (Dako), and muscarinic acetylcholine receptor M3 (Bioss). Biotinylated secondary antibodies, a Vectastain elite ABC kit and a diaminobenzidine substrate kit (Vector) were used to examine the sections under bright-field microscopy to determine the β-cell mass. Alexa Fluor 488- and 555-conjugated secondary antibodies (Invitrogen) were used for fluorescence microscopy analysis. All images were captured using a BZ-9000 microscope (Keyence). The percentage area of the pancreatic tissues occupied by β cells was calculated using BIOREVO software (Keyence)^31^. In the BrdU staining experiment, 22–78 islets in each mouse were analysed to assess the frequency of BrdU-positive cells among the insulin-positive cells.

### Cell culture and treatment

MIN6 cells (kindly provided by Professor Jun-ichi Miyazaki, Division of Stem Cell Regulation Research, Osaka University, Graduate School of Medicine, Osaka, Japan) were used between passages 25 and 40 at ∼80% confluence. The MIN6 cells were grown in Dulbecco’s modified Eagle medium (DMEM), containing 25 mM glucose supplemented with 10% (v/v) heat-inactivated fetal bovine serum (FBS), and equilibrated in 5% CO_2_ and 95% air at 37 °C^32^. Prior to the experiments, the medium was removed and the cells were washed twice with phosphate-buffered saline. Complete details of each treatment are provided in the corresponding figure legend.

### BrdU incorporation in islets and MIN6 cells

Islets were isolated from 8-week-old mice using a method described previously^31^. The isolated islets were cultured in RPMI 1640 medium (Wako Pure Chemical Industries) containing 5.6 mM glucose supplemented with 1% FBS. Following incubation of the islets for 24 h in medium containing 1 mM bethanechol, 0.5 mM carbachol and BrdU, BrdU incorporation was measured with a BrdU cell proliferation assay (Calbiochem) in accordance with the manufacturer’s protocol.

BrdU incorporation was also investigated in the MIN6 pancreatic β-cell line using the FITC-BrdU Flow kit (BD Pharmingen) in accordance with the manufacturer’s protocol. Cells were cultured with serum-free DMEM containing 5.5 mM glucose for 24 h prior to stimulation. Cells were stimulated with muscarinic agonists for 24 h in the same medium, followed by the addition of BrdU. The cells were fixed 20 h later, stained with FITC-BrdU antibodies, and 20,000 cells were analysed by flow cytometry (FACS Canto II, BD Biosciences). The proportion of BrdU-positive cells was calculated using FACS DIVA software (BD Biosciences).

### Immunoblotting

For immunoblotting, >100 isolated islets were lysed in ice-cold radioimmunoprecipitation buffer containing a complete protease inhibitor cocktail (Roche Diagnostics). The lysed islets were subjected to centrifugation and the extracts were subjected to immunoblotting with antibodies to the following proteins: Akt, phospho-Akt, forkhead transcription factor (FoxO1) (Cell Signalling Technology); cyclin D2, forkhead box protein M1 (FoxM1) (Santa Cruz); pancreatic and duodenal homeobox 1 (PDX1) (Merck); or β actin (Abcam). Densitometry was performed using Multi Gauge V3.0 software (FujiFilm).

### Statistical analyses

All data are expressed as the mean ± standard error. All statistical analyses were performed using JMP 11.2.0 software. The data were analysed using Student’s t-tests or analysis of variance. A *P* value < 0.05 was considered to indicate a statistically significant difference.

## Supplementary information

Supplementary information
