# Supplementary material for: The beneficial effects of a muscarinic agonist on pancreatic β-cells
Source: Sci Rep. 2019 Nov 7;9:16180. doi: 10.1038/s41598-019-52691-8 (PMC6838462; doi:10.1038/s41598-019-52691-8)
Supplement: Supplementary file 1 — Supplementary information [file 41598_2019_52691_MOESM1_ESM.pdf]

Supplementary information

Supplementary Table, Figure S1-S6

The beneficial effects of a muscarinic agonist on pancreatic β-cells

Yuzuru Ito, Mitsuyo Kaji, Eri Sakamoto and Yasuo Terauchi

Supplementary Table

Cholinergic side effects of bethanechol

HFD feeding (Body weight)	5-6 week (28.9 ± 0.6 g)	16 week (43.2 ± 1.2 g)
salivation	41.6%	100.0 %
lacrimation	25.0%	33.3 %

A high-fat diet (HFD) was started in 7-week-old C57BL/6J and the experiments were performed after 5-6 weeks or 16 weeks of HFD feeding.
Bethanechol (Bch) 5 μg/g was administered subcutaneously. The number of mice which showed salivation and lacrimation was counted.(5-6 weeks: n=12, 16 weeks: n=6). The data represent percentages.

Supplementary Figure S1

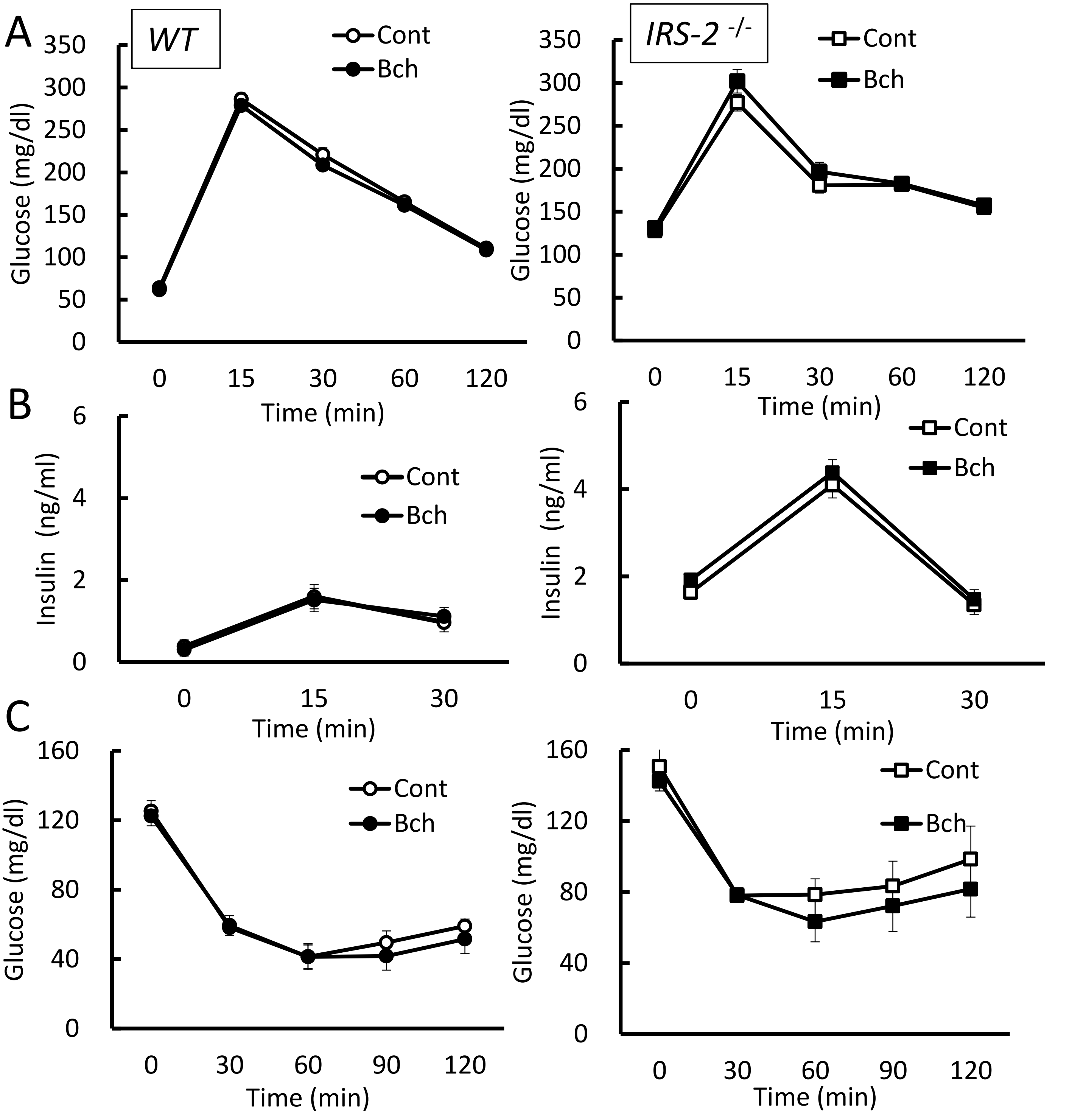

Supplementary Figure S1.
Chronic bethanechol administration in drinking water did not improve glucose tolerance.
The experiments were performed in 8-week-old wild-type (WT) mice and IRS-2 -/- mice. A, Plasma glucose levels during the OGTT after 30 μg/mL bethanechol (Bch) administration in drinking water for 6 weeks. The mice were starved and Bch administration was stopped 20 h before oral glucose loading (1.5 mg/g body weight) in WT mice (left) and IRS-2 -/- mice (right) (n=20–30).
B, Serum insulin levels during the OGTT after 30 μg/mL Bch administration in drinking water for 6 weeks in WT mice (left) and IRS-2 -/- mice (right) (n=15–20).
C, Plasma glucose levels during the insulin tolerance test after 30 μg/mL Bch administration by drinking water for 6 weeks in wild-type mice (left) and IRS-2 -/- mice (right) (n=7–9). The data represent the mean ± SEM; *, $P < 0.05$ vs control.

Supplementary Figure S2

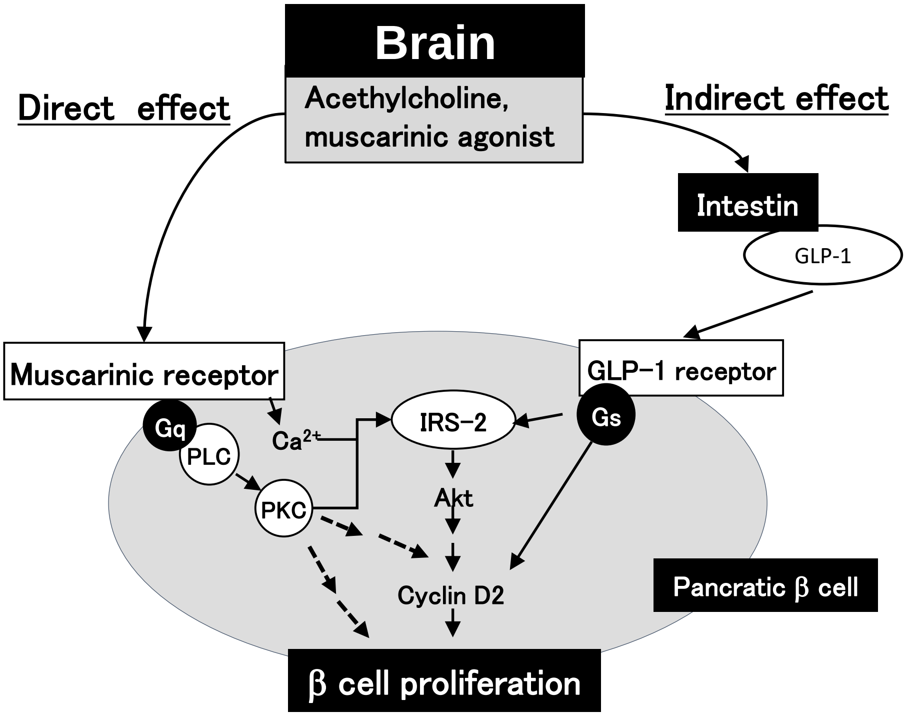

Supplementary Figure S2
A scheme of β-cell proliferation induced by the nervous system.
A signal from the brain is transmitted to many organs through the efferent nerve. The pancreatic β-cell receives the brain signal directly and indirectly. Acetycholine released from the nerve endings directly causes β-cell proliferation via the muscarinic receptor. Acetycholine also stimulates GLP-1 release from the intestine, and GLP-1 acts indirectly on the β-cell. Simultaneous activation of these receptors on the β-cell efficiently promotes β-cell proliferation.

Supplementary Figure S3

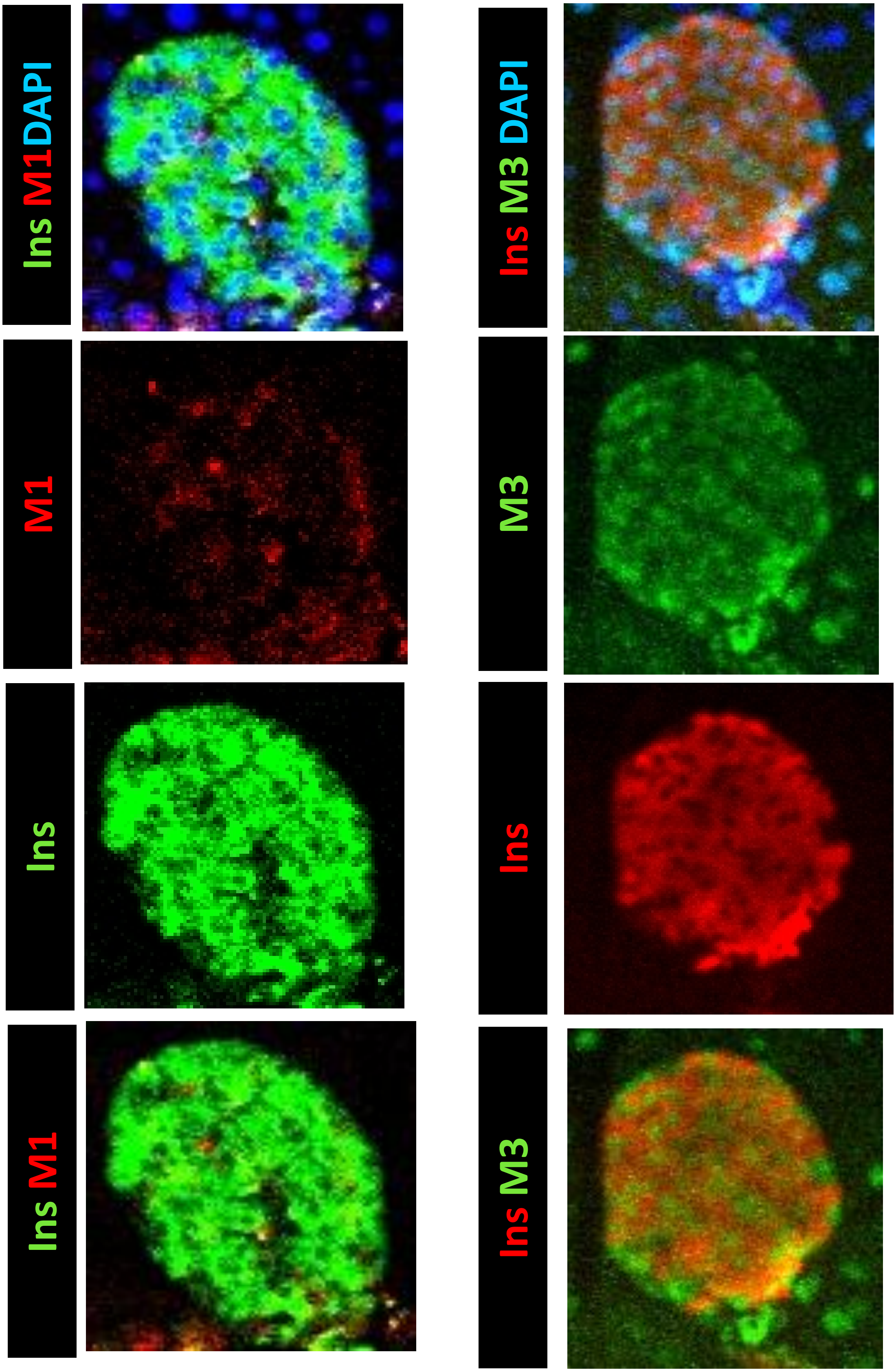

Supplementary Figure S3
Localisation of M1 receptor or M3 receptor in islets from C57BL/6J mice.
Ten-week-old mice were orally administered Bch 10 μg/g once per day for 2 weeks.
Left panel: Insulin is stained green, nuclei are stained blue, and M1 is stained red.
Right panel: Insulin is stained red, nuclei are stained blue, and M3 is stained green.

Supplementary Figure S4

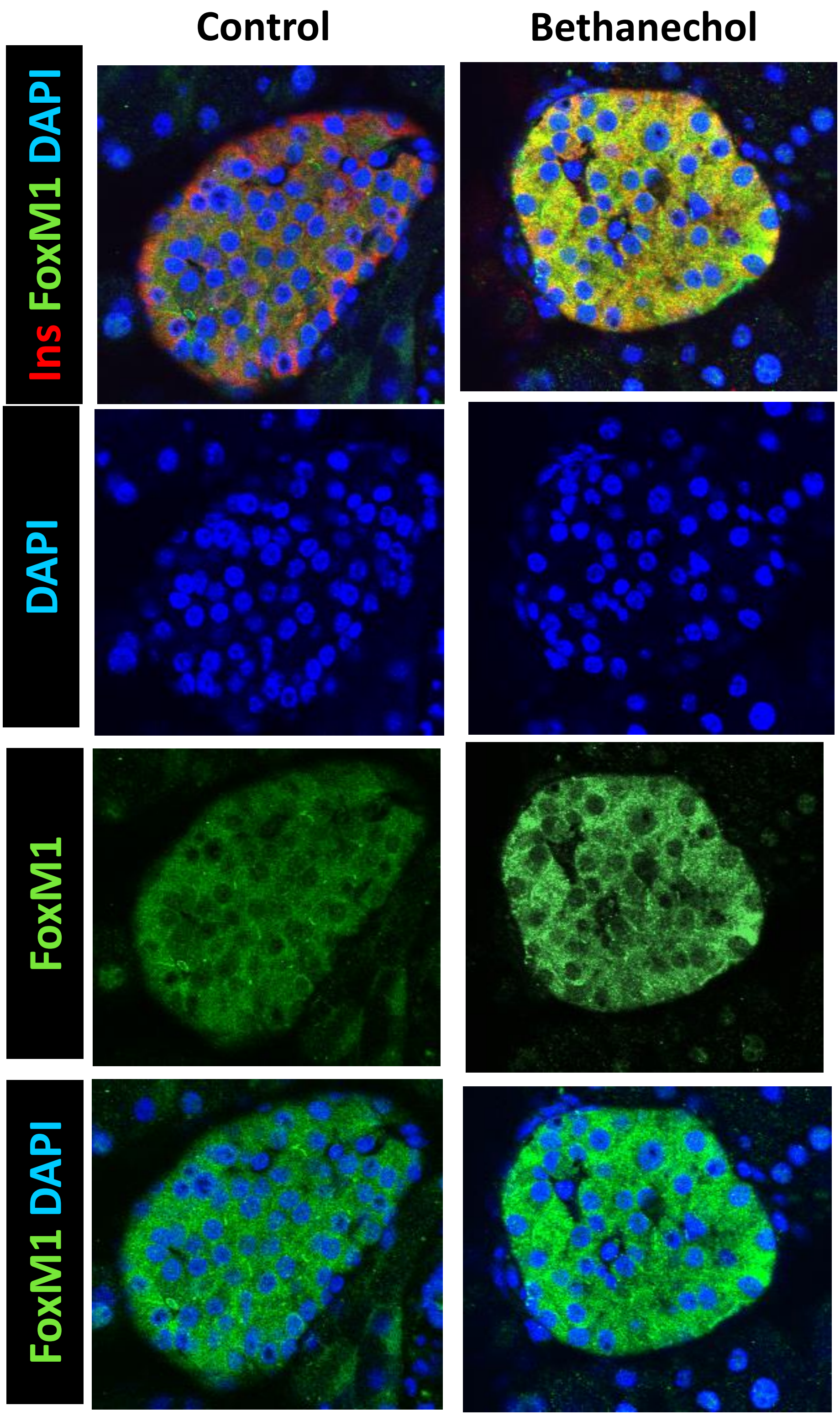

Supplementary Figure S4
FoxM1 localization in islets from bethanechol-administered WT mice.
Eight-week-old WT mice were orally administered 1 μg/g Bch once per day for 2 weeks. Insulin is stained red, nuclei are stained blue, and FoxM1 is stained green.

Supplementary Figure S5

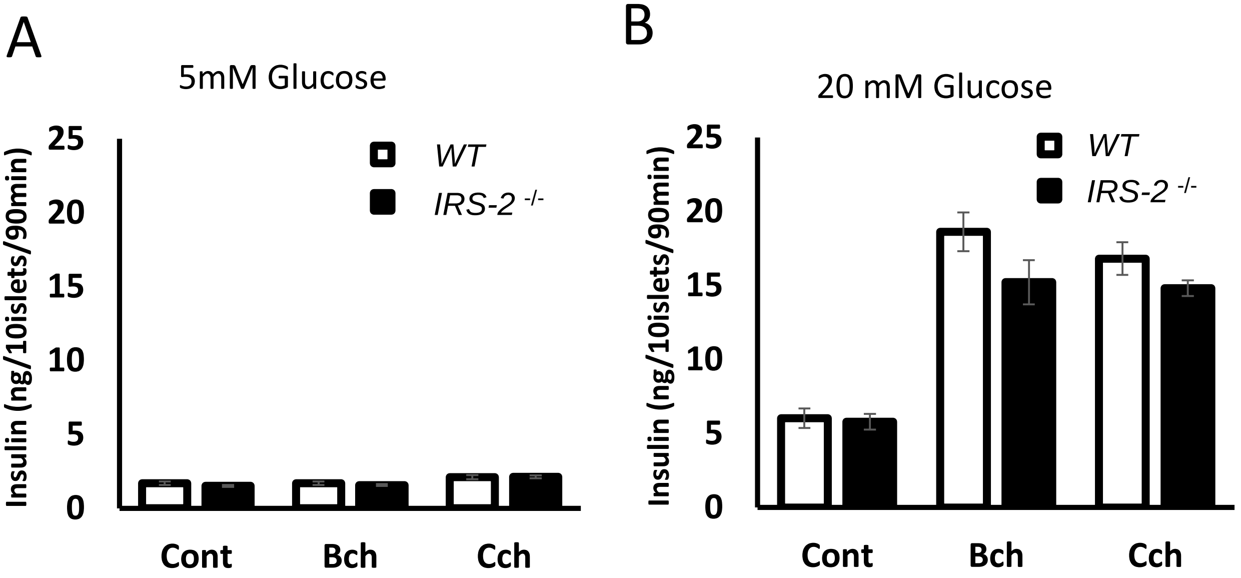

Supplementary Figure S5

Bethanechol enhanced glucose-stimulated insulin secretion under high glucose condition in isolated islets from wild-type mice and *IRS-2* $^{-/-}$ mice.

Islets were isolated from 8-week-old WT mice and *IRS-2* $^{-/-}$ mice by a previously described method (16). Isolated islets were cultured in RPMI 1640 medium containing 5.6mM glucose supplemented with 10% FBS. Ten islets were incubated for 1.5 h in Krebs-Ringer bicarbonate buffer containing 5 or 20 mM glucose, with or without 1mM bethanechol, carbachol (n=4-8). Insulin concentration in the assay buffer was measured with an insulin ELISA kit.

Supplementary Figure S6

Fig. 5A

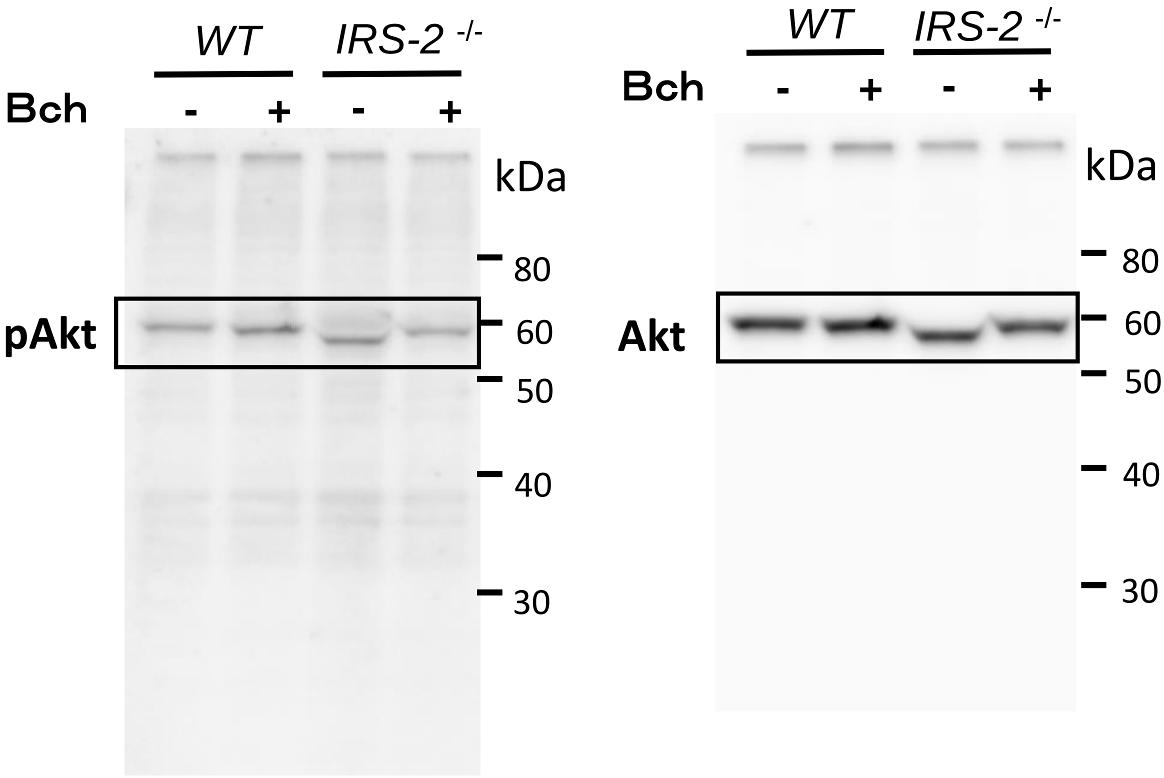

Fig. 5B

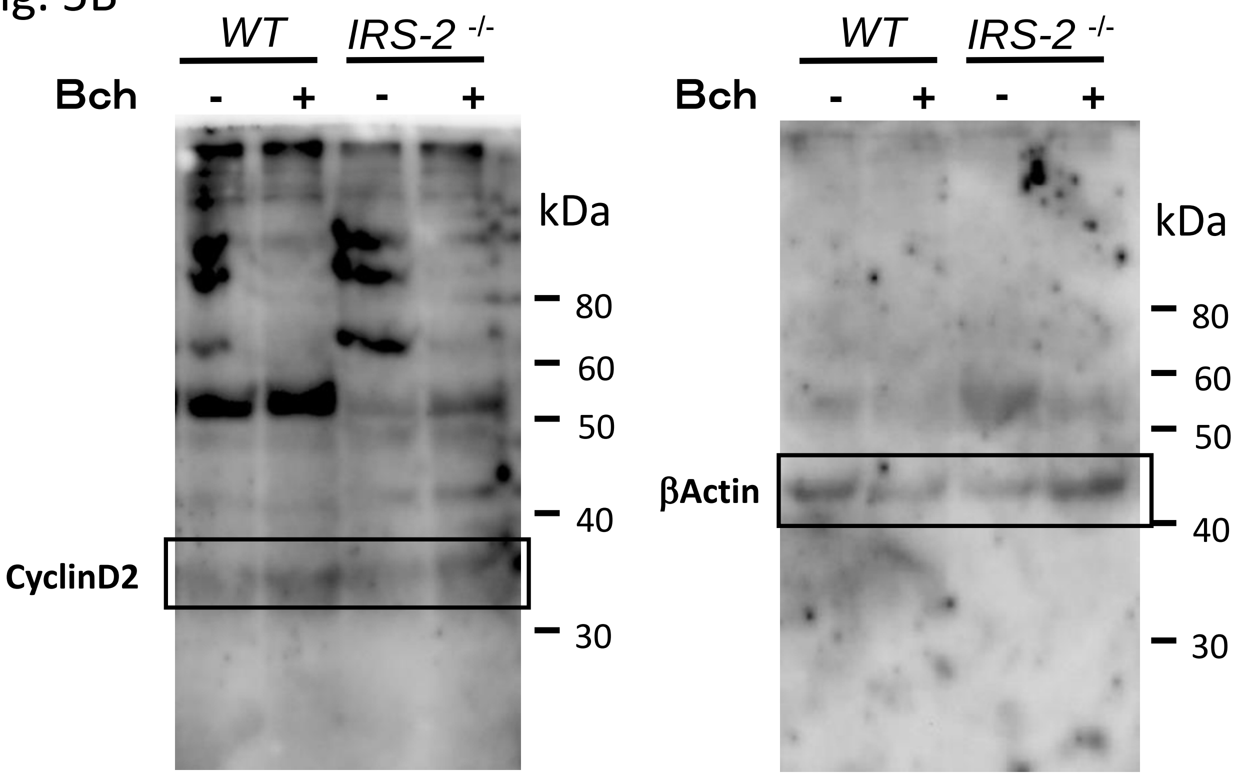

Supplementary Figure S5. Uncropped images of key panels in main figure 5A and 5B. Black boxes indicate the cropped portion of each immunoblot.

Supplementary Figure S6

Fig. 5C

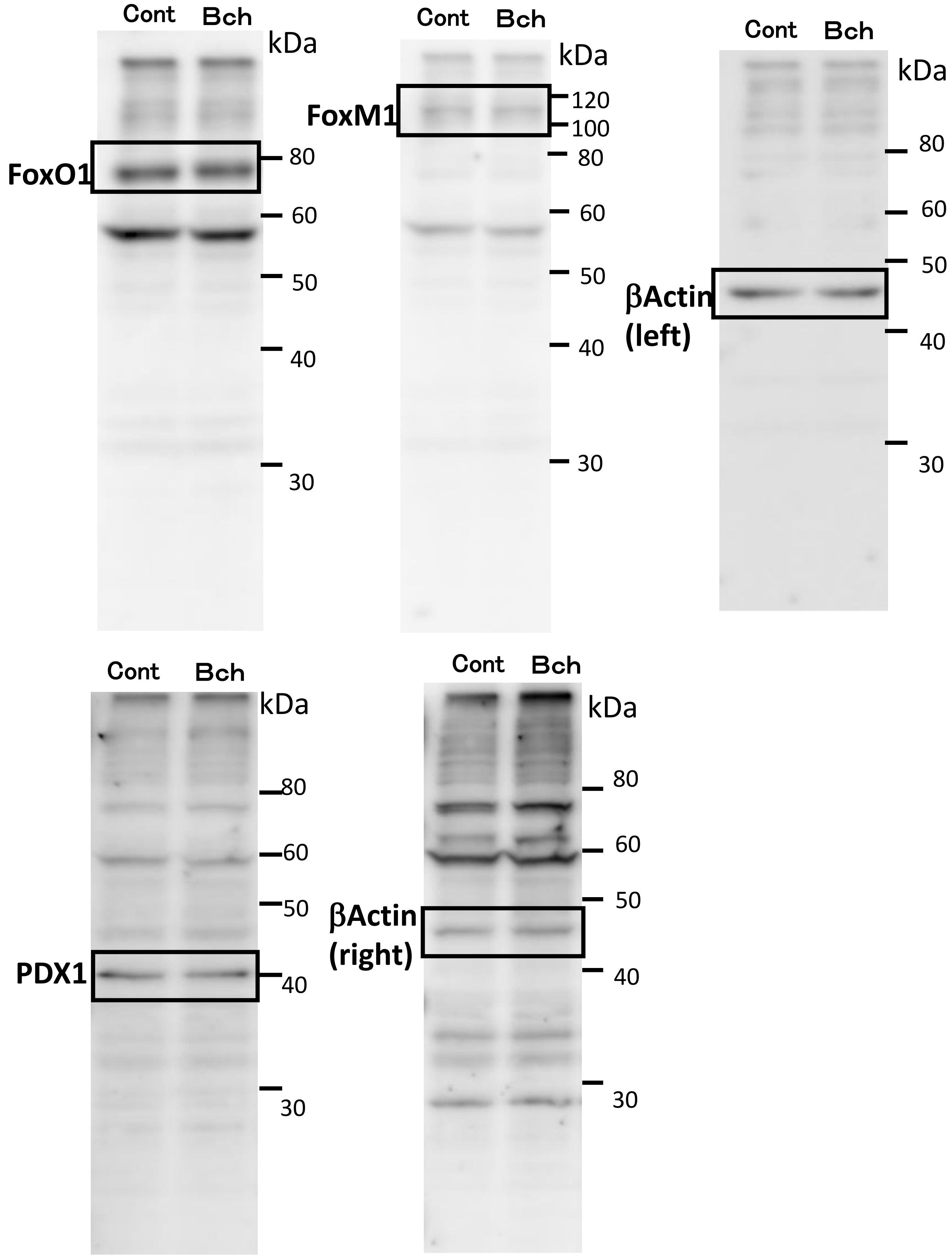

Supplementary Figure S5. Uncropped images of key panels in main figure 5C. Black boxes indicate the cropped portion of each immunoblot.
